# Laparoscopic nephrectomy for giant ruptured renal angiomyolipoma in tuberous sclerosis: case report and literature review

**DOI:** 10.3389/fsurg.2025.1614661

**Published:** 2025-10-21

**Authors:** Yuan Cao, Yougang Liao, Jiao Yan, Kai He, Decai Wang, Yaodong wang, Tiansheng Zhang, Jianjun Wang, Shichun Jiang

**Affiliations:** 1 Department of Urology, School of Medicine, Mianyang Central Hospital, University of Electronic Science and Technology of China, Mianyang, China; 2 Department of Pathology, School of Medicine, Mianyang Central Hospital, University of Electronic Science and Technology of China, Mianyang, China; 3 National Health Commission (NHC) Key Laboratory of Nuclear Technology Medical Transformation, School of Medicine, Mianyang Central Hospital, University of Electronic Science and Technology of China, Mianyang, China; 4 Department of Hepatobiliary Surgery, School of Medicine, Mianyang Central Hospital, University of Electronic Science and Technology of China, Mianyang, China

**Keywords:** tuberous sclerosis complex, renal angiomyolipoma, laparoscopic unilateral nephrectomy, case report, literature review

## Abstract

**Introduction:**

Tuberous sclerosis complex (TSC) is an autosomal dominant disease affecting multiple organs and systems throughout the body. The rupture and bleeding of TSC-associated renal angiomyolipoma (RAML) are the most common causes of death in adults with TSC. Clinically, interventional embolization and open surgery remain the standard treatment options for RAML, with laparoscopic procedures being comparatively less utilized.

**Methods:**

This article details the management of a 59-year-old male with TSC who presented with spontaneous rupture and hemorrhage of a large right renal RAML exceeding 15 cm in diameter. A laparoscopic right nephrectomy was successfully performed as the definitive treatment.

**Results:**

The laparoscopic procedure was successful in managing the acute hemorrhage and removing the affected kidney. The patient's postoperative course was uncomplicated.

**Discussion:**

The diagnostic and therapeutic management of this complex case is discussed, along with a review of the relevant literature. This case demonstrates that laparoscopic nephrectomy can be a viable surgical option for the emergency treatment of giant TSC-associated RAML rupture and bleeding in selected patients.

## 1 Introduction

Tuberous sclerosis complex (TSC), also known as Bourneville disease, is an autosomal dominant systemic disease characterized by multi-organ misshapen lesions (1). It is caused by the abnormal activation of the mammalian target of rapamycin (mTOR) signaling pathway owing to mutations in the TSC1 and/or TSC2 genes, and involves multiple organs and systems throughout the body, especially the kidney, brain, skin, and heart. In general, renal lesions, epilepsy, and cardiac rhabdomyosarcoma are the common causes of death in patients with TSC (2). Renal angiomyolipoma (RAML) are present in approximately 80% of patients aged > 10 years (3). These lesions comprise malformed blood vessels, smooth muscle, and adipose tissue components (4). The management of ruptured TSC-associated RAML primarily includes interventional embolization, medical therapy, and surgical intervention. Selective arterial embolization can rapidly achieve hemostasis while preserving renal function, though it carries risks such as postoperative pain, recurrent bleeding, and tumor recurrence.mTOR inhibitors, such as everolimus, can reduce tumor volume and decrease the risk of bleeding; however, they require long-term administration and are associated with adverse effects including immunosuppression (25). Surgical intervention is indicated for patients with large tumors or active hemorrhage: nephron-sparing surgery (NSS) allows maximal preservation of renal function but is technically challenging and carries potential risks such as delayed bleeding and urinary leakage; radical nephrectomy is reserved for cases with severe renal impairment, albeit at the expense of permanent loss of kidney function.When technically feasible, laparoscopic surgery provides distinct advantages, including minimal invasiveness, enhanced intraoperative visualization, and accelerated postoperative recovery. Treatment strategies should be individualized according to tumor features and patient-specific factors.We report a case of laparoscopic nephrectomy for the rupture and bleeding of a giant TSC-associated RAML, summarize the available literature, and retrospectively analyze the clinical diagnosis of the disease, available treatment options, and prognosis.

## 2 Case description

**Figure 1:**
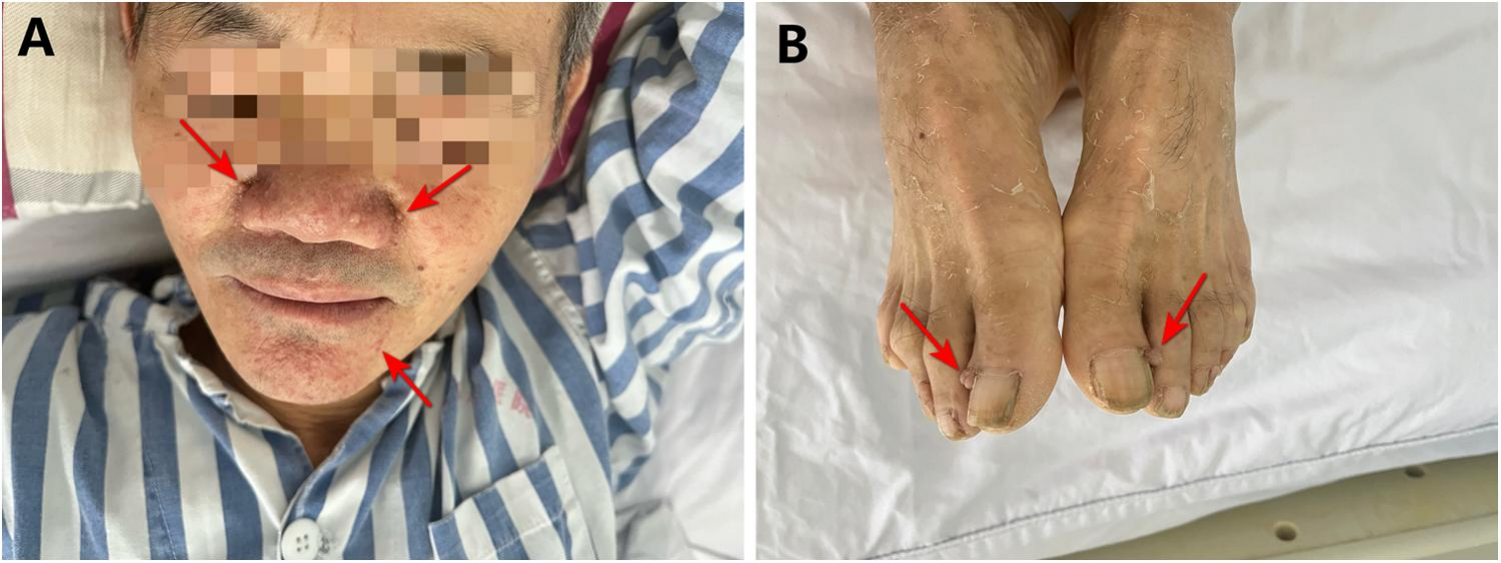
Clinical presentation of the patient. **(A)** Angiofibroma of the face. **(B)** Periungual fibroma of the bunion on both lower extremities.

**Figure 2:**
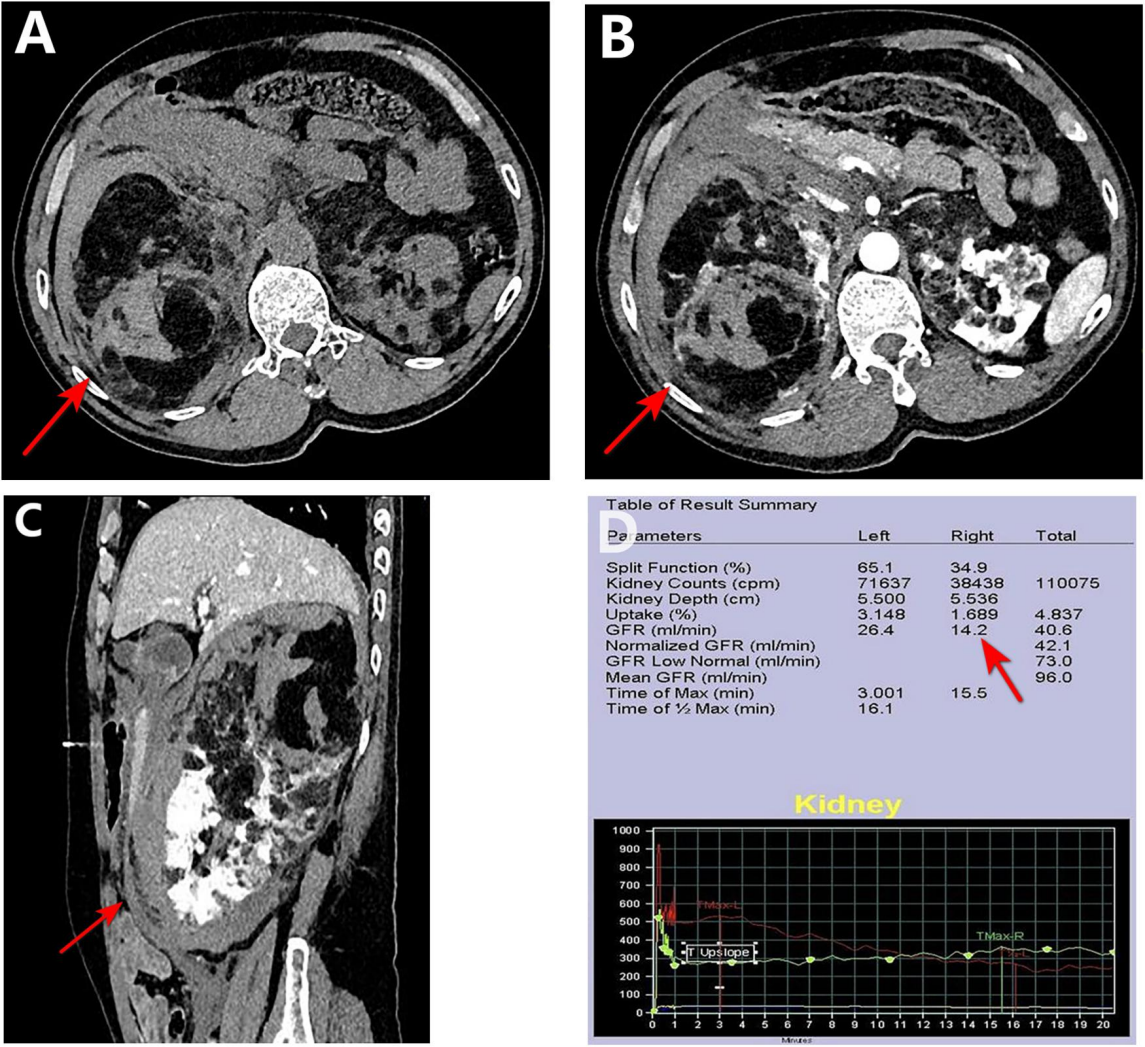
Preoperative CT and renal function. **(A–C)** CT images show a ∼17.5 cm mass (arrows) in the right kidney with hemorrhage and poor enhancement. **(D)** GFR measurement confirms markedly reduced right kidney function.

The patient was a 59-year-old man who was admitted to the emergency room with “right upper abdominal distension and pain for 4 h”. He denied any history of epileptic seizures and his personal, familial, or marital history were unremarkable, and his intelligence was normal. His temperature, heart rate, respiratory rate, blood pressure, oxygen saturation, and body mass index were 36.7°C, 81 beats/min, 23 breaths/min, 155/89 mmHg (1 mmHg = 0.133 kPa), 98%, and 23.4 kg/m^2^, respectively. The skin at the bilateral nasolabial folds showed a butterfly-shaped distribution of dark red papules; around the mouth, nose, and lower lip, we observed a dark red papule with a size ranging from a grain of rice to a green bean, exhibiting a hard texture. The nails of both toes were rough and thick, and a mung bean-sized fibroma was observed on the nail bed of the bunion of each lower limb (Figure 1). Abdominal examination: palpable mass in the right abdomen, slightly hard, right abdominal muscle tension, obvious pressure pain, no obvious rebound pain, and positive percussion pain in the right kidney area. Laboratory results on admission were as follows: white blood cell count, red blood cell count, platelet count, hemoglobin level, and blood creatinine of 7.61 × 10^9^/L, 3.12 × 10^12^/L, 56 × 10^9^/L, 95 g/L, and 72.7 umol/L, respectively. The results of liver and kidney function, electrolyte levels, coagulation, and urinalysis were within normal limits. Enhanced abdominal computed tomography (CT) showed multiple vascular smooth muscle lipomas in both kidneys, with rupturing of the right kidney. Scattered blood accumulation in the surrounding renal peritoneum, abdominal cavity, and retroperitoneum should be considered in combination with other clinical findings. The largest tumor was located in the right kidney, with a size of 13.3 cm × 10.2 cm × 17.5 cm. Glomerular filtration rate (GFR) measurement: both kidneys were impaired, with the right kidney being significantly impaired; the values of the left and right kidneys were: 26.4 and 14.2 ml/min, respectively. Based on the patient's clinical records and biochemical parameters and abdominal CT findings, the initial diagnosis was benign bilateral renal tumors with rupture and hemorrhage of a large tumor in the right kidney. Given the substantial tumor dimensions and persistent hemorrhagic risk confirmed through preoperative assessment, laparoscopic right nephrectomy was conducted (Figure 2).

**Figure 3:**
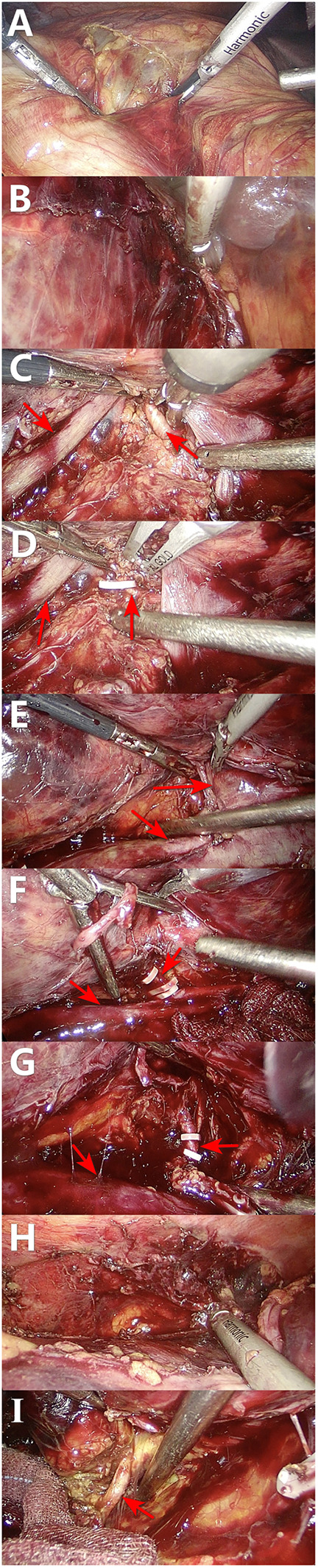
Intraoperative sequence of laparoscopic right nephrectomy. **(A,B)** Initial mobilization along the white line of Toldt and division of the hepatocolic ligament. **(C,D)** The right renal artery (red arrow) is isolated between the inferior vena cava (white arrow) and aorta, and controlled with clips. **(E,F)** The right renal vein is similarly exposed and ligated. **(G)** The renal artery is fully skeletonized and transected. **(H)** The kidney is mobilized by lysing perirenal adhesions. **(I)** The ureter is identified, clipped, and divided.

On the third day after admission, a laparoscopic right nephrectomy was performed under general anesthesia. Intraoperative exploration showed that: the right kidney was significantly enlarged in size and morphology; a hematoma was observed in the renal fat capsule; the tumor pushed out the inferior vena cava and duodenum and densely adhered to the surrounding tissues, which were congested and edematous; and the trauma was clearly oozing blood. During the procedure, we separated the greater omentum from the abdominal adhesions, incised the peritoneum along Toldt's line, dissociated the hepatic colonic ligament, separated it along Toldt's gap, revealed the inferior vena cava, isolated the right renal artery between the inferior vena cava and abdominal aorta, and blocked the right renal artery using one medium-sized hem-o-lok clip. Subsequently, the right renal vein was ligated using three large hem-o-lok clips. The right renal artery was further blocked using two medium-sized hem-o-lok clips, and after dissecting the right renal artery, the perirenal adherent tissues were adequately freed. Moreover, after separating the right kidney and perirenal fat capsule together with the periphery, the ureter was located in the plane of the lower pole of the right kidney, distally separated as far as possible, and ligated. The entire operation lasted 145 min with intraoperative bleeding of approximately 200 ml. The patient recovered well after the operation, and for three consecutive days, the drainage from the right renal fossa was approximately 100 ml/d, and the color of the drainage fluid (ascites) was light red. On the fifth postoperative day, the blood analysis showed that the hemoglobin and creatinine levels were 87 g/L and 113.8 umol/L, respectively, and no obvious abnormality was observed in the liver function and electrolytes. Subsequently, the patient was discharged from the hospital after administering everolimus at 10 mg/d, and after 2 months of everolimus the patient stopped on his own (owing to financial difficulties). The patient was evaluated in the outpatient clinic for 6 months and did not exhibit any significant discomfort (Figure 3).

**Figure 4:**
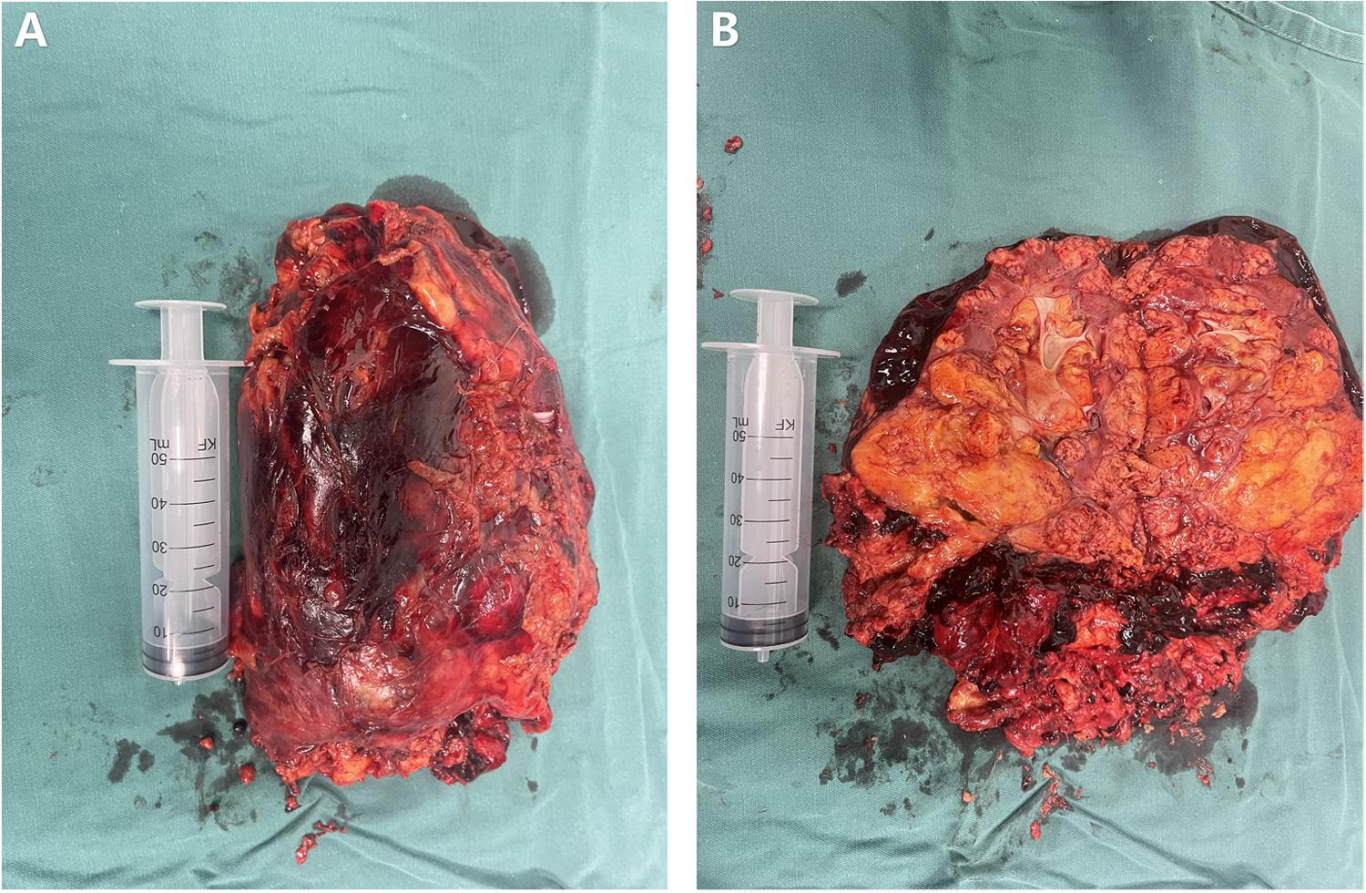
Postoperative specimen of the patient. **(A)** Postoperative isolated specimen of the right kidney. **(B)** Dissected postoperative isolated specimen of the right kidney.

**Figure 5:**
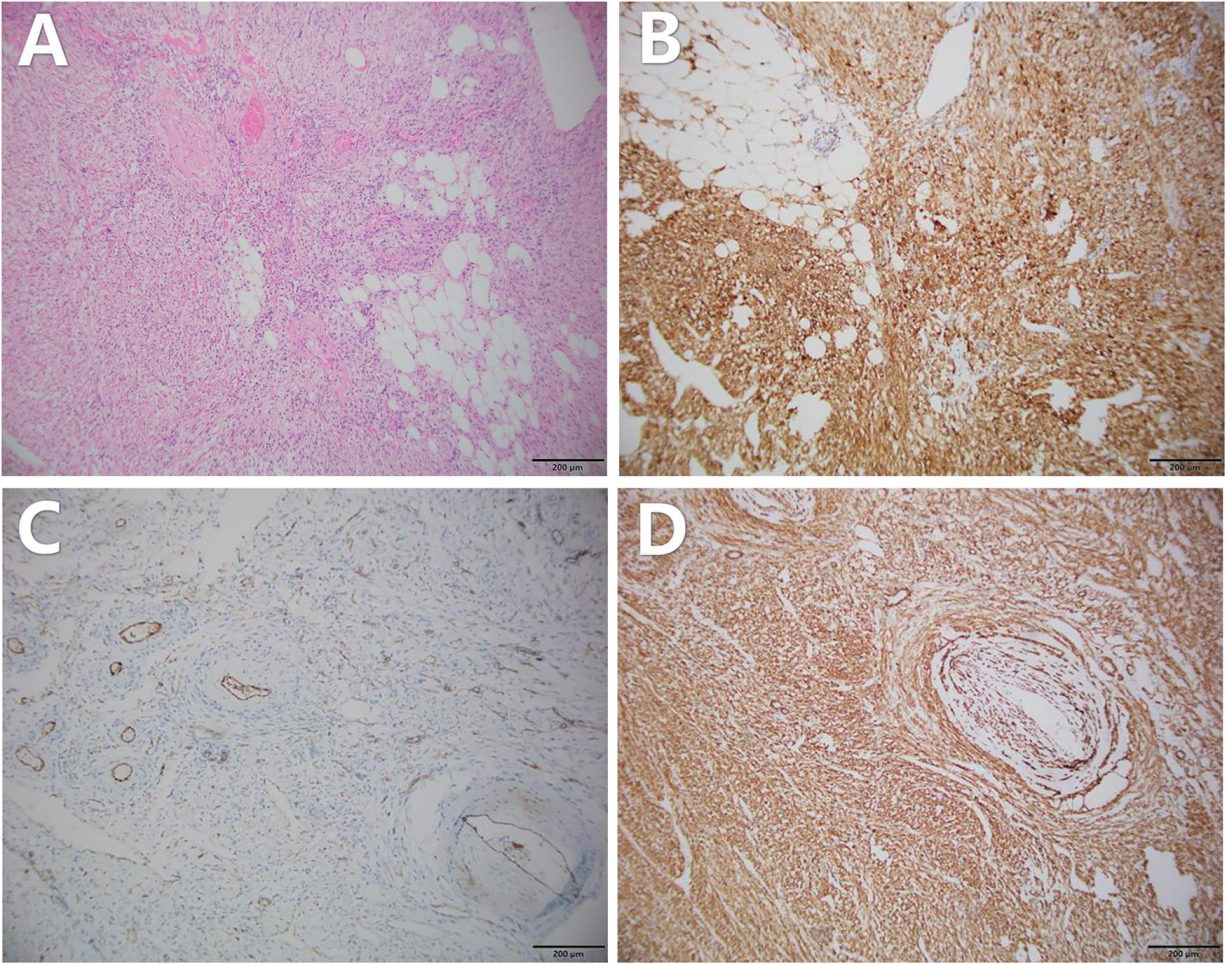
Pathology of the patient's postoperative specimen. **(A)** HE staining shows admixture of vessels, smooth muscle, and fat. Immunohistochemistry suggests the following: **(B)** MART-1 positivity, **(C)** CD34 (vascular+), and **(D)** SMA positivity. The above pathology images were magnified at ×100.

The postoperative specimen (right renal tumor) suggests renal tissue with sizes of 22 cm × 21 cm × 5.5 cm and 12.5 cm × 10.1 cm × 7.5 cm. We observed a gray-yellow mass in section and gray-yellow solid medium in section. It was focally necrotic, and its mass occupies the entire renal section. No normal renal structures were observed; ureteral dissection was 5.5 cm in length and tubular meridian was 0.3 cm in length (Figure 4). Pathological findings suggest (right renal tumor) spindle cell tumor with long spindle shaped cells, abundant cytoplasm, and an eosinophilic tumor, with adipocytes and thick-walled blood vessels in the background. Immunohistochemical results: CD117 (−), CA IX (−), PAX2 (−), PAX8 (−), HMB45 (foci +), MART-1 (+), CD34 (vascular +), SMA (+), TFE3 (−), and Ki-67 (+, approximately 1%) (Figure 5). The combination of pathological examination and immunohistochemical findings confirmed the final diagnosis of RAML.

## 3 Discussion

TSC manifests as multi-systemic misshapen tumors, characterized by misshapen tumors in multiple organ systems, such as the skin, brain, heart, lungs, retina, and kidneys (5). A clinical diagnosis can be made based on clinical manifestations, and genetic diagnosis is feasible in patients who cannot be clinically diagnosed. In this report, the patient was diagnosed with TSC owing to the presence of more than two major features: periungual fibroma, facial angiofibroma, and RAML, highlighted by a ruptured hemorrhage of the RAML. RAML occurs in more than 80% of patients (6). It is more common in females than in males, is overwhelmingly bilateral, and has a family history in approximately 1/3 of patients. In this case, the patient did not have a familial genetic disease, and a mutation in the *de novo TSC1* or *TSC2* gene, which encodes a dysfunctional misfolded tumor protein and nodulin, possibly caused the abnormal proliferation and differentiation of the histiocytes, leading to the development of TSCs. TSC-associated RAML is a highly vascularized benign tumor comprising blood vessels, smooth muscle, and adipose tissue. Although its pathological features resemble those of sporadic RAML, this variant is characterized by several distinct manifestations: 1. multicentric and bilateral involvement; 2. a prominent smooth muscle component, frequently accompanied by cellular atypia; and 3. an increased likelihood of containing epithelioid components with malignant potential.These characteristics contribute to a more aggressive clinical behavior in TSC-associated RAML, including accelerated growth, an elevated risk of hemorrhage, and a necessity for lifelong monitoring due to the potential for recurrence and malignant transformation (7). Its early stages may have no clinical manifestations, and with the development of the disease, patients may develop abdominal mass, abdominal pain, urinary tract infection, chronic renal insufficiency, hypertension, and hemorrhage, among other symptoms. In severe cases, acute rupture and hemorrhage may be complicated, causing perirenal or retroperitoneal hematoma, and even leading to hemorrhagic shock and acute renal failure, which are life-threatening (8).

To better contextualize the clinical relevance of the present case, a systematic retrieval and synthesis of previously reported cases of giant TSC-associated RAML was performed (Table 1). The literature review included studies published up to April 2025. The search was conducted using the PubMed database with the following key terms: tuberous sclerosis complex, renal angiomyolipoma, diagnosis, treatment, and prognosis. Comparative analysis indicates that for tumors of this considerable size, particularly those complicated by rupture and hemorrhage, conventional management has predominantly relied on open nephrectomy or staged intervention following emergency embolization (9, 12, 21). This preference is largely attributable to concerns regarding intraoperative hemorrhage control and the challenges posed by severe adhesions in laparoscopic settings. Nonetheless, the successful outcome in this case provides valuable clinical insight into alternative approaches. Compared with the cases reported by Winterkorn and Kodati, direct laparoscopic nephrectomy in this instance obviated the risk of subsequent RAML enlargement and decreased the necessity for additional treatment (10, 22). Although the tumor size was similar to that documented by the Arachchi group (16.3 cm), the present procedure was completed entirely laparoscopically and did not require intensive care unit admission, underscoring the feasibility and safety of this approach when performed by a proficient surgical team (14). In contrast to the two-stage surgical strategy following embolization as described by Parekh and Bellouki (12, 21), the single-stage laparoscopic radical resection performed herein circumvented the risks associated with multiple interventions and delayed rebleeding, offering the patient a definitive “one-stop” therapeutic solution. The successful application of this technique, however, critically depends on meticulous preoperative evaluation, early control of the renal vasculature, and advanced laparoscopic operative skills.

**Table 1:** Previous reports of TSC-associated RAML with treatment measures and outcomes.

Ref.	Sex	Diagnostic approaches	Size, cm	side	Symptoms	Operation	Outcomes
(9)	F	IVU + CT	45	L	Left flank pain	RON	The patient was no significant change in the size of the right kidney at one year follow up
(10)	M	US + MR	8.4	L	NM	embolization	MR at the 10-month follow-up showed an interval increase with no symptoms
(11)	M	US	13.5	R	acute abdominal pain	Selective angioembolization	Follow-up one year period, no complications were observed.
(12)	F	CT	29 (R) NM (L)	B	abdominal distension and flank pain	embolization + RON	The patient was stable at follow-up
(13)	F	CT	26	L	bilateral dull aching flank pain, more on left side	LON	The patient was asymptomatic at six months follow up
(14)	M	CT	16.3	L	left iliac fossa pain and macroscopic haematuria	LON	three years later, dyspnoea worsened secondary to lung metastasis
(15)	F	CT	6.1	L	Left lower back pain	Embolization	The patient was stable at six months follow up
(16)	M	CT	NM	B	right abdominal pain	embolization	Eight months of outpatient follow-up showed no recurrence
(17)	F	CT	17	R	hematuria and right flank pain	Selective angioembolization	The patient was stable at 18 days after the procedure
(18)	M	US + CT	35(R) 14(L)	B	right abdominal swelling	NM	NM
(19)	F	CT	NM	B	Left flank pain and hematuria	Selective angioembolization	The patient was discharged 5 days later without complications
(20)	M	US + CT	8	R	Intermittent bilateral flank pain	angioembolisation	Following the procedure, flank pain resolved completely
(21)	F	CT	25	L	progressive asthenia and abdominal pain	embolization + RON	The postoperative course of this patient was uneventful, with no complications
(22)	F	CT	23(R) 25(L)	B	Right flank pain associated with hematuria	coil embolization	Clinical improvement was rapid and discharge was possible with oral sirolimus 2 mg/day

F, female; M, male; CT, computed tomography; MR, magnetic resonance; US, ultrasound; IVU, intravenous pyelogram; NM, not mentioned; L, left; B, both; R, right; RON, right open nephrectomy; LON, left open nephrectomy.

To differentiate TSC-associated RAML from renal malignancy, magnetic resonance imaging (MRI) is recommended as the imaging test of choice for its diagnosis and follow-up evaluation. In this case, the patient was admitted to the hospital as an emergency for right renal rupture and bleeding, and the patient's financial condition was average; thus, CT examination was employed for diagnosis. Additionally, the patient's blood pressure should be monitored as the risk of hypertension increases with the severity of renal lesions. The patient's postoperative pathology suggests that the right nephrectomized tissue can be observed as angiomyolipoma with smooth muscle lipomas; and multinucleated cells are observed in the foci, which can be a diagnosis of renal staggering tumors. The presence of multinucleated cells in the foci does not indicate renal malignant tumors, and without any obvious abnormality on immunohistochemistry analyses, and the patient should be closely evaluated after the operation.

The primary goals for patients with TSC-associated RAML are to avoid bleeding, control the size of the RAML, and preserve renal function. Primary treatments for this disease include active monitoring, medication, embolization, and surgical removal. Surgery is not routinely recommended as TSC-associated RAML is bilateral and multiple surgeries can lead to a decline in renal function. mTOR inhibitors can reduce tumor size and decrease the risk of secondary rupture bleeding in patients with high-risk TSC-associated RAML (23). mTOR inhibitors are recommended as the first line of therapeutic options according to the National Comprehensive Cancer Network (NCCN) guidelines (24). However, the problem with long-term therapy with the mTOR inhibitor everolimus is that it reduces the size of tumors but does not cure them, thereby increasing the chances of gonadal dysfunction, interstitial lung disease, and immunosuppression-related complications (25). Selective renal artery embolization is currently an effective treatment for ruptured TSC-associated RAML hemorrhages, especially in patients with acute hemorrhage or those undergoing high-risk surgery. Selective renal artery embolization is currently the preferred treatment for TSC-associated RAML tumors ≥4 cm in diameter with clinical symptoms and bleeding tendency, especially in patients with retroperitoneal hemorrhage. Selective renal artery embolization is minimally invasive, rapidly hemostatic, safe, and effective, ensuring the maximum protection of normal renal tissue. Its disadvantage is a high recurrence rate of 24% (26) and the need to combine it with pharmacological and local ablative therapies. Associated complications, such as post-embolization syndrome, acute renal failure, ectopic embolization, and infection, may also occur. Nephrectomy or surgery with the preservation of the renal unit may be an option for suspected malignancies, large tumors, and those with a high risk of bleeding. Preoperative imaging revealed a massively ruptured tumor with active hemorrhage, indicating extensive destruction of the renal parenchyma. This assessment was confirmed by the postoperative specimen, which showed near-total replacement of renal architecture by the lesion, with no identifiable normal structures and only minimal residual functional nephrons. Radical resection enabled rapid and complete removal of both the bleeding source and necrotic tissue, thereby resolving the condition in a single procedure. This strategy averted potential complications associated with embolization and eliminated the need for reoperation. Partial nephrectomy represents an ideal nephron-sparing approach for smaller, peripherally located tumors; however, its feasibility is highly contingent upon specific anatomical features. In the present case, the massive size of the tumor, combined with complete rupture and active hemorrhage, resulted in extensive adhesions to adjacent tissues and effacement of normal anatomical planes.Attempting NSS under such conditions entails substantial risks, such as uncontrollable intraoperative bleeding and subsequent complications including urinary leakage and hemorrhage. The technical feasibility in this scenario is considerably limited.

However, there are few reports on the use of laparoscopic surgical resection for ruptured TSC-associated RAML hemorrhages with a diameter >15.0 cm, and practical experience is relatively lacking. Laparoscopic surgery provides benefits including minimal invasiveness, enhanced intraoperative visualization, and accelerated postoperative recovery. However, its limitations—such as restricted visual field that may necessitate conversion to open surgery, and challenges in hemorrhage control that increase the risk of substantial blood loss—must be carefully considered. In this case, the considerable tumor size may impede intraoperative identification of the renal artery, particularly for ruptured and bleeding tumors, making renal artery blockade challenging. Intraoperative blockage is crucial for tumor resection owing to the richer blood supply to the tumor. In this case of laparoscopic right nephrectomy, finding the renal artery between the abdominal aorta and inferior vena cava for blockage may be feasible.

Selective renal artery embolization was not considered the treatment of choice in the treatment strategy, and laparoscopic unilateral nephrectomy was administered directly, with a high risk of postoperative complications of renal impairment. The treatment strategy did not adhere to the guidelines but did not significantly affect renal function in the postoperative period, and the prognosis was good. The patient's unilateral lesion was severe, with a rich blood supply, and selective renal artery embolization may lead to re-bleeding owing to collateral circulation or incomplete embolization. Embolization may increase the difficulty of subsequent surgery, such as tissue adhesion and changes in the anatomical structure. The rupture and bleeding of the TSC-associated RAML in the patient after the review of blood routine suggested that the hemoglobin stabilized at approximately 95 g/L, and the condition was relatively stable. The preoperative preparations can be further improved; the effect of embolization of the tumor is limited and it cannot cure the disease. Long-term follow-up evaluation is required, and the patient's economic conditions in this case were limited. Thus, he did not undergo embolization, and direct laparoscopic unilateral nephrectomy was conducted. This case study will be of reference significance for urologists.

## 4 Conclusion

Patients with ruptured or bleeding giant TSC-associated RAML require early surgical intervention. Laparoscopic unilateral nephrectomy is safe and feasible after rigorous preoperative preparation.

## Data Availability

The original contributions presented in the study are included in the article/Supplementary Material, further inquiries can be directed to the corresponding author/s.
